# Respiratory adenovirus infections in children: a focus on Africa

**DOI:** 10.1097/MOP.0000000000001335

**Published:** 2024-02-29

**Authors:** Marieke M. van der Zalm, Nadia A. Sam-Agudu, Lilly M. Verhagen

**Affiliations:** a Desmond Tutu TB Centre, Department of Paediatrics and Child Health, Faculty of Medicine and Health Sciences, Stellenbosch University, Cape Town, South Africa; b International Research Center of Excellence, Institute of Human Virology Nigeria, Abuja, Nigeria; c Department of Pediatrics and Child Health, University of Cape Coast School of Medical Sciences, Cape Coast, Ghana; d Global Pediatrics program and Division of Infectious Diseases, Department of Pediatrics, University of Minnesota Medical School, Minneapolis, MN, USA; e Department of Pediatric Infectious Diseases and Immunology, Amalia Children's Hospital; f Department of Laboratory Medicine, Laboratory of Medical Immunology, Radboud Center for Infectious Diseases, Radboud University Medical Center, Nijmegen, Netherlands

**Keywords:** *Adenoviridae* infections, Africa, child, lung, pneumonia

## Abstract

**Purpose of review:**

Lower respiratory tract infections (LRTIs) are an important cause of child morbidity and mortality globally, especially in children under the age of 5 years in Africa. Respiratory viruses, including human adenoviruses (HAdVs), are common causes of LRTIs in children. This review aims to shed light on the epidemiology, clinical manifestations, sequelae, and treatment options specific to adenovirus respiratory infections in African children.

**Recent findings:**

Recent evidence has challenged the perception that adenovirus is a negligible cause of LRTIs. Studies show HAdV emerging as the third most common viral pathogen in fatal pneumonias among under-5 children in low-income and middle-income African countries, contributing to 5.5% of all pneumonia deaths and ranking second in hospital-associated viral pneumonia deaths. Predominant HAdV serotypes associated with disease differ by country and region, and have changed over time. Risk factors for increased disease severity and long-term respiratory sequelae in previously healthy African children with HAdV LRTIs are not well established.

**Summary:**

Although respiratory viruses, including HAdV, are recognized contributors to LRTIs, the prevalence and impact of adenovirus infections have been under-recognized and understated. Available data suggests that African children, particularly those under 5 years old, are at risk of severe sequelae from respiratory HAdV infections. Long-term sequelae, including bronchiectasis and postinfectious bronchiolitis obliterans, further underscore the significant impact of HAdV infections. However, the scarcity of comprehensive data hampers our understanding of the extent of the impact of HAdV infections on child lung health in Africa. We recommend scaled-up HAdV surveillance, ensuring its consistent inclusion in population-level LRTI assessments, and expanded and equitable access to diagnostics for early recognition of African children at risk of developing chronic sequelae and death. Enhanced understanding of adenovirus epidemiology and clinical outcomes and the availability of therapeutic options are essential for informed public health strategies and clinical care.

## INTRODUCTION

Lower respiratory tract infections (LRTIs) remain an important cause of morbidity and mortality globally, especially among young children in Africa [1,2]. Respiratory viruses are a major cause of acute respiratory illness and LRTIs in children. A recent systematic review of the cause of childhood pneumonia in low-income and middle-income countries (LMICs) between 2010 and 2020 showed that respiratory syncytial virus (RSV), human metapneumovirus (HMPV), influenza, parainfluenza, *Streptococcus pneumoniae*, *Haemophilus influenzae*, *Staphylococcus aureus*, *Mycoplasma pneumoniae,* and *Mycobacterium tuberculosis* accounted for most pneumonia cases globally [3^▪^]. However, the most common causative pathogens varied by age; human adenovirus (HAdV) was among the most common pathogens in children under five years of age.

HAdVs are not often highlighted as a major cause of pneumonia, but they are frequently linked to long-term respiratory sequelae [4–6]. A recent large postmortem study in under-5 children across six African countries and one South Asian country showed pneumonia as implicated in the causal pathway in 40% of deaths, with adenovirus being the third most common viral pathogen, occurring in 5.5% of all pneumonia deaths [7^▪▪^]. HAdV was also implicated in almost 13% of hospital-associated pneumonia deaths, ranking as the second most prevalent virus behind cytomegalovirus, but more frequent than RSV. This shows that adenovirus pneumonia is not only more prevalent in African countries than commonly acknowledged, but also underscores its association with significant mortality.

HAdVs are ubiquitous in human environments and are a relatively common source of infection. Owing to their immunological immaturity, children are disproportionately affected by HAdV infections. HAdV was first isolated from adenoid tissue of young American children in 1952–1953 [8]. More recently, in 2022, HAdV serotype 41F was suggested to be associated with reported cases of rare but severe acute hepatitis of unknown origin, affecting over 1000 young children in the United Kingdom, the United States, and several countries in other regions [9–12].

In this review, we present an up-to-date overview of the epidemiology, clinical manifestations, sequelae and treatment options for pediatric respiratory HAdV infections, with a focus on African children under 18 years of age. 

**Box 1:**
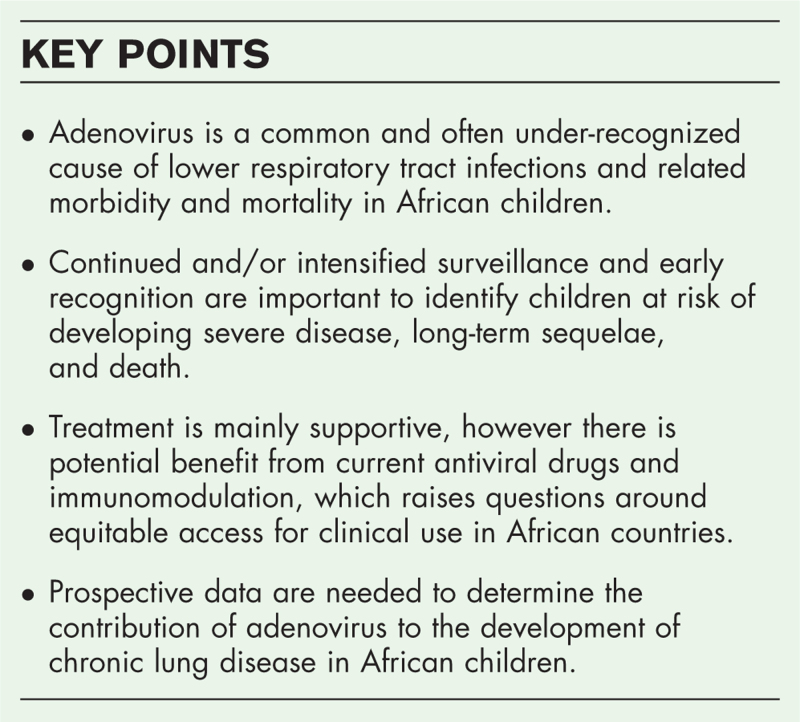
no caption available

## EPIDEMIOLOGY

Although HAdV is considered an all-year virus, its prevalence differs between the northern and southern hemispheres [13,14]. Most studies on HAdV epidemiology have been conducted during outbreaks and/or in specific populations, such as hospitalized children or immunocompromised individuals who are more susceptible to HAdV infection [14,15]. Although the contribution of HAdV pediatric respiratory infections is well documented in high- income countries, limited data are available from African countries. A recent systematic review reported on the prevalence of respiratory viruses in 32 African studies, of which 14 studies from nine countries included data on HAdVs [16^▪▪^]. HAdVs were identified as causative agents in 1–31% of respiratory infections. Predominant HAdV serotypes associated with disease differed by country and region, and changed over time. In a South African study, HAdV was among the top three most commonly detected viruses in both medically and nonmedically attended influenza-like illness and severe respiratory illness [17]. Although the highest rates of severe respiratory illness because of HAdV were seen in children less than 5 years of age, the rates of influenza-like illness because of HAdV were highest among individuals aged 5–24 years, indicating that HAdV impacts young people in addition to young children in Africa [17]. In another South African study, HAdV was detected in 55% of nasopharyngeal aspirates collected in children presenting with presumptive pulmonary tuberculosis [18].

## TRANSMISSION

HAdV transmission is mainly by direct person-to-person spread through infected respiratory tract secretions, fomites, and aerosols [14]. Contact with contaminated food, water, or surfaces contribute to feco-oral routes of transmission. While outbreaks have followed exposure at swimming pools, summer camps, childcare centers and in healthcare settings, there is limited information on the extent to which crowding increases HAdV transmission and disease in densely populated or more crowded settings [19]. Considering the propensity for outbreaks in over-crowded settings, there have been suggestions of a risk for HAdV modifications through recombinations between circulating HAdV (serotypes 3 and 7 in particular) and zoonotic AdV, with pandemic potential [19].

Careful caregiver hand hygiene and proper (e.g. bleach-based) disinfection of instruments and objects in the environment remain primary tools for preventing adenovirus spread in home, group childcare, and healthcare settings [20]. Alcohol, detergents, and chlorhexidine do not eradicate adenoviruses from hands or equipment because these viruses are non-enveloped and are, therefore, resistant to these agents. Consequently, HAdV has been less impacted by the implementation and easing of public health measures during the COVID-19 pandemic: a Zambian study reported HAdV being identified at higher rates from nasopharyngeal samples throughout the 2020-2021 period of the COVID-19 pandemic, compared to before the pandemic [21]. Interestingly, this heightened presence of adenovirus was observed a year into the pandemic rather than in its early stages [22], suggesting a nuanced temporal dynamic that may be related to the virus gradually occupying a specific ecological niche [23,24].

## PATHOPHYSIOLOGY AND IMMUNE RESPONSES

### Viral subtypes and classification

Adenoviruses are relatively small, nonenveloped double-stranded DNA viruses of the genus Mastadenovirus, family *Adenoviridae*. To date, there are more than 100 known types that can infect a variety of hosts and all vertebrates, including humans, mammals, birds, fish and reptiles. Adenoviruses are classified into seven subgroups, A–G. This classification was originally based on their neutralization phenotypes (serotypes), while genomic characteristics (genotypes) have later been added [25]. There is evidence for persistent adenovirus circulation in nonhuman primates in Africa, and gorilla-to-human transmission has been described for group C [26].

The subgroups most associated with respiratory tract disease are B (serotypes 3 and 7), C (serotypes 1, 2, and 5) and E (serotype 4). The most severe LRTIs are because of adenovirus types B7, and to a lesser extent, B3, which can cause necrotizing pneumonia [27]. Recently, HAdV-B55 has also been associated with outbreaks of pneumonia in both immunocompetent and immunocompromised children [28]. Although initially identified in respiratory isolates from Asia, this strain has subsequently spread globally, including to the African region.

The most prevalent serotypes differ across countries or regions. For example, HAdV-B3 was most common in mainly under-5 Ugandan outpatients with influenza-like illness [29], whereas HAdV-C was most common in hospitalized children in Tunisia [30].

### Innate and adaptive immune responses

HAdV entry into the cell triggers a rapid response from various innate immune signaling pathways, causing the production and release of proinflammatory cytokines and chemokines. Initial innate immune responses in the airway mucosal epithelia are particularly important because these tissues are specifically targeted by HAdV and present the first line of defense against infection. For example, the receptor for advanced glycation end products (RAGE) is constitutively expressed in lung tissue and immune cells and is a key factor in the initiation of the inflammatory response to HAdV infection. RAGE signaling results in the initiation of inflammation, which is subsequently blocked by its soluble form, sRAGE. Patients with HAdV infections exhibiting decreased sRAGE were more likely to develop pneumonia, likely as a result of excessive inflammation and severe tissue injury [31].

Innate immunity supports the activation and proliferation of antigen-specific adaptive immune responses against HAdV. CD8^+^ T cells produce proinflammatory cytokines such as interferon gamma (IFN-ɣ) and tumor necrosis factor (TNF), and also kill infected cells presenting viral peptides bound to MHC I via the release of perforin and granzyme-containing granules, or by inducing apoptosis. However, hyperfunction of CD8^+^ T cells producing excessive levels of IFN-ɣ might be related to disease severity in children [32]. B cells produce high-affinity, type-specific antibodies against the HAdV capsid proteins. Anti-HAdV antibodies can target HAdV for opsonization, activate the classical complement pathway, neutralize viral particles and induce inflammasome activation in cells internalizing opsonized virus.

HAdV seroprevalence is lowest in children under 5 years of age, whereas most adults will be sero-positive for at least one serotype [33]. Prolonged asymptomatic viral shedding from the respiratory tract can occur postrecovery, viral shedding in hospitalized children with severe HAdV pneumonia in China lasted approximately 51 and 97 days in HAdV-B3 and HAdV-B7-positive infections, respectively [34]. It has been suggested that prolonged viral shedding could be the source of endemic circulation and epidemic outbreaks [19].

Finally, an open question is whether the increasing use of viral vector vaccines will affect HAdV immunity. Owing to the COVID-19 pandemic, there has been an expansion in the use of adenoviral vector vaccines, with doses given to billions of people worldwide. This may be particularly relevant for vector vaccines based on HAdV-5, given its global circulation. It will be important to evaluate the cross-protection that these vaccines may provide against HAdV-5, and potentially other viral types associated with subgroup C, as this could potentially generate selective pressure for new circulating viral types [19,35].

## CLINICAL PRESENTATION

HAdV is associated with a wide range of diseases ranging from mild upper respiratory tract symptoms to more serious conditions. The tissue tropism of HAdV types varies, and this impacts clinical manifestations of infection. In asymptomatic individuals, the virus can be present in peripheral blood lymphocytes, respiratory tissue, and normal duodenal epithelium [14,36,37]. Beyond upper and LRTIs, HAdV types are associated with gastroenteritis, genitourinary infections, as well as cardiac and neurologic infections. Additionally, patients with disseminated adenoviral disease, including those who are immunocompromised, may experience fulminant hepatic necrosis [38].

HAdV can cause severe and fatal disease in both immunocompetent and immunocompromised hosts, particularly in the pediatric under-5 population [6,39]. Among immunocompromised patients, infection is most frequently described in hematopoietic stem cell transplant recipients and solid organ transplant recipients [15,39]. A South African study comparing LRTI cause in hospitalized under-5 children living and not living with HIV showed a significantly higher proportion of adenovirus infections in children living with HIV (32 vs. 27%, *P* = 0.013) [40]. A more recent South African study showed that up to one-third of 206 children hospitalized with laboratory-confirmed adenovirus pneumonia was malnourished [41]. Other underlying conditions included HIV (7% previously undiagnosed, 20% exposed and uninfected) and other conditions such as cardiac (23%) or respiratory (13%) diseases. HIV exposure, HIV infection and malnutrition were not associated with mortality in this cohort. Strikingly, 37% of these patients needed intensive care, confirming that adenovirus can cause significant morbidity in African children.

### Respiratory sequelae

Long-term respiratory sequelae from viral pathogens such as HAdV have been well documented [5,42]. Up to 55% of children develop long-term respiratory sequelae following adenovirus infection, including bronchiectasis and postinfectious bronchiolitis obliterans [5,6]. Most patients in the previously mentioned South African cohort of 206 children with adenovirus pneumonia recovered completely, but 14% developed persistent lung disease, which was associated with hypoxia and need for intensive care [41]. Additionally, adenovirus was identified as the most common cause of postinfectious bronchiolitis obliterans in a South African cohort of 51 children aged 6 months to 15 years [43].

A systematic review and meta-analysis on the sequelae of childhood pneumonia [5] found 13 articles describing long-term outcomes, of which two articles came from Africa but both without pathogen specification. The exact contribution of HAdV to pulmonary damage is unknown as most data on long-term post-LRTI lung function monitoring in previously healthy children focus on RSV, and most studies come from high-income countries [44,45].

## DIAGNOSTICS

Adenovirus can be detected in several sample types, and the choice of specimen depends on site of infection and clinical presentation. Samples can be tested using different laboratory techniques, including viral culture, serology, antigen test, immunofluorescence assays, and PCR testing. The choice of diagnostic method depends on factors such as availability of resources, urgency of the diagnosis, and factors specific to the sample collected. PCR is often preferred because of its high sensitivity and specificity and short turn-around time for results, even though costs can be high. For respiratory adenovirus infection, respiratory specimens such as nasopharyngeal aspirates, nose and throat swabs, or bronchoalveolar lavage (BAL) samples can be collected for testing. BAL samples are typically regarded as the gold standard for diagnosing LRTIs. Nevertheless, this approach involves an invasive procedure that requires sedation and specialized medical intervention. A recent study from China compared nasopharyngeal swabs (NPS) with BAL samples and found that NPS with a very high viral load were associated with 100% detection of HAdV in BAL samples [46]. This suggests that NPS samples are effective for detecting HAdV and underscores the significance of high viral load measurements in the upper airways, suggesting a strong likelihood of the virus being present in the lungs as well. Furthermore, HAdV is significantly more frequently detected in saliva compared with NPS samples, likely because initial replication of adenovirus occurs in the oropharynx [9]. Saliva samples might, therefore, be suitable for adenovirus screening, and the detection of high viral loads might represent HAdV presence in the lungs as well [47].

## TREATMENT OPTIONS

Treatment of HAdV respiratory disease is primarily supportive [14,48]. Most infections in otherwise healthy children are self-limited and do not require treatment. In children on immunosuppressive treatment with disseminated HAdV infection, reduction of immunosuppressants can be considered, if feasible. If symptoms evolve to severe pneumonia, timely implementation of respiratory support up to extracorporeal membrane oxygenation may improve prognosis.

Intravenous immunoglobulin has been used to treat acute myocarditis, pneumonia (in immunocompromised patients), and disseminated disease due to adenovirus [27,39]. Glucocorticoids have also been used in patients with adenovirus-associated pneumonia; early administration may improve treatment success and reduce inpatient mortality [27,48].

**FIGURE 1:**
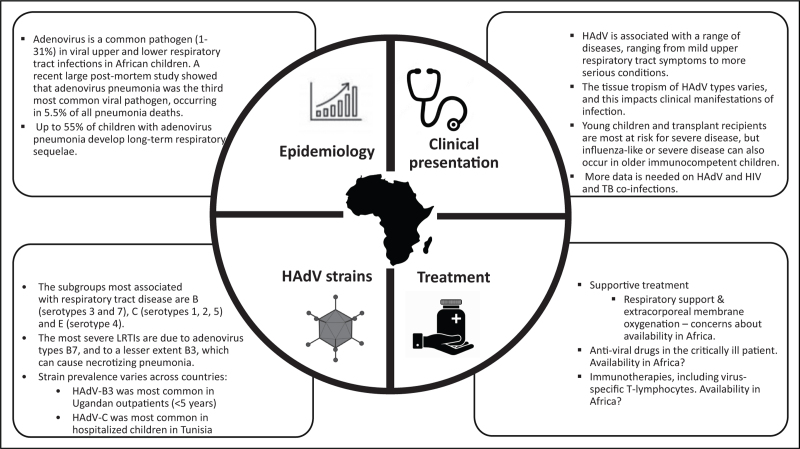
A summary of pediatric respiratory adenovirus data from Africa.

Cidofovir has emerged as the currently preferred antiviral agent for treatment of adenoviral disease, although it is only available intravenously. Cidofovir-related nephrotoxicity and myelotoxicity is a concern, and has been described in high-risk hematopoietic stem cell transplant patients [15,39], However, only a minority of immunocompetent children treated with cidofovir seem to develop nephrotoxicity. Recently, brincidofovir, an oral formulation lipid conjugate of cidofovir, has been reported to have good oral bioavailability, and reduced nephrotoxicity and bone marrow toxicity compared with cidofovir [48]. Other orally bioavailable cidofovir analogues with reduced nephrotoxicity and increased activity are under development [49]. Novel donor-derived virus-specific T-lymphocyte therapies appear to have good efficacy and low toxicity, making them increasingly attractive [50]. Finally, pre-clinical studies of monoclonal antibodies show that these may provide serotype-specific protection for possible use for prophylactic or therapeutic purposes [51]. For such therapies to become available in African LMICs, equity considerations must be made and HAdV must be centered as a contributor to long-term pulmonary sequelae and mortality in African children. Although there is some information on epidemiology and sequelae, the lack of data on preventive and treatment measures among African children is likely to limit equitable access to these interventions (Fig. 1).

## CONCLUSION

Across Africa, HAdV outbreaks generally go unreported or under-reported, largely because of a lack of awareness regarding prevalence, and attributable morbidity and mortality, as well as limited availability of diagnostic tests. Our review highlights HAdV as an important and under-recognized cause of severe pneumonia, persistent lung disease and pneumonia-associated mortality in young African children. The recent COVID-19 pandemic has brought about shifts in the epidemiology of respiratory viruses, and the inherent resistance of HAdVs to common disinfectants may have led to an increase in HAdV infections in some African countries. This finding aligns with the need for continued and/or intensified surveillance, especially among children. In addition, early recognition of severe adenovirus pneumonia is important to identify children at risk of developing chronic sequelae and death, and more prospective data are needed to determine the contribution of adenovirus to the development of chronic lung disease in African children. Finally, it is essential to scale up current and future diagnostics and treatment options for HAdV-associated LRTIs and make them readily available for clinical use in African countries.

## Acknowledgements

*The authors would like to acknowledge clinicians, researchers, health care personnel and child health advocates who work with us to prevent and treat paediatric respiratory infections, and the African children who motivate us to be better at this work.*

### Financial support and sponsorship

*Funding received by NIH, EDCTP, Radboud UMC.*

*M.V.D.Z. is supported by a career development grant from the EDCTP2 program supported by the European Union (grant number TMA2019SFP-2836 TB-Lung FACT2), by the Fogarty International Center (FIC) of the US National Institutes of Health (NIH) under award number K43TW011028 and funding from a researcher-initiated grant from the South African Medical Research Council. N.A.S.A. is supported by the NIH/FIC under award number 5D43TW012280-03 and under a small award for the Central and West Africa Implementation Science Alliance (CAWISA) through the NIH/FIC Adolescent HIV Prevention and Treatment Implementation Science Alliance (AHISA). L.M.V. is supported by a Hypatia Fellowship funded by the Radboud University Medical Center, Nijmegen, The Netherlands.*

### Conflicts of interest

*There are no conflicts of interest.*

## REFERENCES AND RECOMMENDED READING

Papers of particular interest, published within the annual period of review, have been highlighted as:

▪ of special interest
▪▪ of outstanding interest
